# Identification of Aortic Proteins Involved in Arterial Stiffness in Spontaneously Hypertensive Rats Treated With Perindopril:A Proteomic Approach

**DOI:** 10.3389/fphys.2021.624515

**Published:** 2021-02-10

**Authors:** Danyelle S. Miotto, Aline Dionizio, André M. Jacomini, Anderson S. Zago, Marília Afonso Rabelo Buzalaf, Sandra L. Amaral

**Affiliations:** ^1^Joint Graduate Program in Physiological Sciences, Federal University of Sao Carlos and São Paulo State University, UFSCar/UNESP, São Carlos, Brazil; ^2^Department of Biological Sciences, Bauru School of Dentistry, University of São Paulo, Bauru, Brazil; ^3^Post-Graduate Program in Movement Sciences, São Paulo State University, Bauru, Brazil; ^4^Department of Physical Education, School of Sciences, São Paulo State University, Bauru, Brazil

**Keywords:** pulse wave velocity, proteomic analysis, ACE inhibitor, hypertension, aorta artery

## Abstract

Arterial stiffness, frequently associated with hypertension, is associated with disorganization of the vascular wall and has been recognized as an independent predictor of all-cause mortality. The identification of the molecular mechanisms involved in aortic stiffness would be an emerging target for hypertension therapeutic intervention. This study evaluated the effects of perindopril on pulse wave velocity (PWV) and on the differentially expressed proteins in aorta of spontaneously hypertensive rats (SHR), using a proteomic approach. SHR and Wistar rats were treated with perindopril (SHR_P_) or water (SHRc and Wistar rats) for 8 weeks. At the end, SHR_C_ presented higher systolic blood pressure (SBP, +70%) and PWV (+31%) compared with Wistar rats. SHR_P_ had higher values of nitrite concentration and lower PWV compared with SHR_C_. From 21 upregulated proteins in the aortic wall from SHR_C_, most of them were involved with the actin cytoskeleton organization, like *Tropomyosin* and *Cofilin-1*. After perindopril treatment, there was an upregulation of the *GDP dissociation inhibitors* (*GDIs*), which normally inhibits the *RhoA/Rho-kinase/cofilin-1* pathway and may contribute to decreased arterial stiffening. In conclusion, the results of the present study revealed that treatment with perindopril reduced SBP and PWV in SHR. In addition, the proteomic analysis in aorta suggested, for the first time, that the *RhoA/Rho-kinase/Cofilin-1* pathway may be inhibited by perindopril-induced upregulation of *GDIs* or increases in NO bioavailability in SHR. Therefore, we may propose that activation of *GDIs* or inhibition of *RhoA/Rho-kinase* pathway could be a possible strategy to treat arterial stiffness.

## Introduction

Arterial stiffness has been recognized as an independent predictor of all-cause mortality, not only in population with diseases like hypertension, diabetes, and renal disease (Hamilton et al., 2007; Sakuragi and Abhayaratna, 2010; Vlachopoulos et al., 2010), but also in overall population (Mitchell, 2014; Nilsson Wadstrom et al., 2019; Scuteri et al., 2020). It is well established that hypertension, associated or not with aging, leads to increased arterial stiffness (Morgan et al., 2014; Phillips et al., 2015; Lindesay et al., 2016, 2018; Lacolley et al., 2017; Rode et al., 2020), assessed by pulse wave velocity (PWV), even though some authors have shown that the development of arterial stiffness may be prior to hypertension (Celik et al., 2006; Kaess et al., 2012), which may cause increases in afterload and left ventricular remodeling (Zieman et al., 2005; Ohyama et al., 2016).

Assessment of PWV has been performed in humans using ultrasound, Doppler, magnetic resonance imaging, and applanation tonometry techniques (Mitchell et al., 2010; Phillips et al., 2015; Ohyama et al., 2016; Obeid et al., 2017a, b; Nilsson Wadstrom et al., 2019; Rode et al., 2020; Scuteri et al., 2020); although human studies precisely determine the compliance and arterial stiffness via the dynamic properties of the arterial wall, they are limited in advancing our knowledge on conditioning mechanisms. The possibility of having an experimental model with the measurement of both blood pressure (BP) and PWV, simultaneously with direct access to the arteries for gene, protein, histological studies, and other assays, represents an important advancement for better understanding the mechanisms involved in arterial stiffness changes. In this regard, our group recently standardized a new non-invasive device for assessment of arterial stiffness in rats (Fabricio et al., 2020) and showed that it is able to detect changes in arterial stiffness that are conditioned by age- and pressure-related arterial remodeling.

Aortic stiffening is associated with either a remodeling or disorganization of the vascular wall, which derives from an increased collagenous material, fibrotic components, the presence of elastin fiber fracture, arterial elasticity, or vascular smooth muscle cell (VSMC) hypertrophy (Morgan et al., 2014; Lindesay et al., 2016, 2018; Fabricio et al., 2020; Steppan et al., 2020). Among several causes of aortic stiffness, the central role of the renin–angiotensin system (RAS) is well known, and therefore, some studies have shown the effects of RAS inhibition on arterial stiffness (Marque et al., 2002; Gonzalez et al., 2018) and, consequently, on BP, but the exact molecular mechanisms induced by RAS on aortic stiffness are not completely understood.

Since either hypertension may induce arterial stiffness or arterial stiffness may induce hypertension, understanding the mechanisms involved in aortic stiffness would be an emerging target for therapeutic intervention to prevent and/or treat hypertension. Thus, the aim of this study was to evaluate the effects of perindopril treatment on PWV and to identify the differentially expressed proteins in the aorta of spontaneously hypertensive rats (SHR), using a proteomic approach.

## Materials and Methods

Twenty-two SHR (250–300 g, 3 months) and ten Wistar rats (similar age) were obtained from the Animal Facility of Institute of Biomedical Sciences, University of São Paulo, (USP) and São Paulo State University (UNESP), campus of Botucatu, SP, Brazil, respectively. All rats were housed at the animal facility maintenance at School of Sciences, São Paulo State University—UNESP, campus of Bauru. All rats received water and food (Biobase, Águas Frias, SC, Brazil) *ad libitum* and were maintained in a dark–light cycle (12–12 h) in a controlled temperature room (22 ± 2°C). All methods used were approved by the Committee for Ethical Use of Animals at School of Sciences, UNESP (#778/2017 vol. 1).

### Pharmacological Protocol

The animals were separated into three groups with similar body weight (BW) and randomly assigned to undergo an experimental protocol through 8 weeks: SHRc (*n* = 12): SHR treated daily with tap water; SHR_P_ (*n* = 10): SHR treated daily with perindopril; and Wistar (*n* = 10): Wistar rats treated daily with tap water.

During the experimental protocol, rats were treated daily with perindopril, an angiotensin II-converting enzyme inhibitor (Conversyl^®^, 3 mg/kg of BW), or tap water, *via gavage*, at 9 a.m. for 8 weeks. This dose was chosen based on previous publication (Yazawa et al., 2011). In order to test the effectiveness of the pharmacological treatment, a bolus of Angiotensin I was infused after treatment period (100 μl, at dose of 1 μg/μl, i.v.) in two treated and two control rats and AP response was evaluated.

### Functional and Biochemical Analyses

#### Pulse Wave Velocity

After 60 days of pharmacological treatment, the assessment of PWV was performed as previously published (Fabricio et al., 2020). In summary, each rat was anesthetized with xylazine hydrochloride (Anasedan^®^, 10 mg/kg) and ketamine hydrochloride (Dopalen^®^, 50 mg/kg), and two pOpet^®^ probes (Axelife SAS, Saint Nicolas de Redon, France) were positioned on the right forelimb (close to elbow) and hindlimb (close to knee). After stabilization of the signal (in a quiet room), the transit time (TT, ms) was recorded for 10 s and registered by pOpet 1.0 software. Taking together the travelled distance (D, cm), estimated by the distance between the two probes, and TT, the PWV was calculated using the following formula:

PWV(m/s)=D(m)/TT(s)

For PWV analysis, 10 measurements of each rat were done and the average was calculated.

#### Blood Pressure Measurements

Systolic blood pressure (SBP) was measured every other week during the experimental protocol using a tail-cuff plethysmography system (PanLab LE5001, Barcelona, Spain). Before the experimental protocol, each rat was subjected to an adaptation period in the restraint cage (5 days before). For the measurement, each rat was allocated into the restraint cage, which was preheated at 37°C. Keeping the rat into the restraint cage, a cuff was positioned around the tail of the rat (outside of the cage), just before the transducer, which detected tail arterial pulse. Systolic BP (through tail-cuff technique) was determined when the first pulse was detected during the deflating process. Rat’s tail BP was considered as the mean of five measurements (Amaral et al., 2000).

At the end, 24 h after PWV assessment, all rats were anesthetized with xylazine hydrochloride (Anasedan^®^, 10 mg/ml) and ketamine hydrochloride (Dopalen^®^, 50 mg/kg) from Ceva Sante Animale, Paulínea, SP, Brazil, and the carotid artery was catheterized, as previously published (Amaral et al., 2000). After 24-h recovery, pulsatile pressure of each awake animal was continuously recorded for at least 1 h, in a quiet room, using a pressure transducer (DPT100, Utah Medical Products Inc., Midvale, UT, United States) connected to the artery cannula, which sent the signal to an amplifier (Quad Bridge Amp, ADInstruments, NSW, Australia) and then to an acquisition board (Powerlab 4/35, ADInstruments, NSW, Australia), as previously published (Duchatsch et al., 2018). SBP was derived from pulsatile BP recordings, using a computer software (Labchart pro v7.1, ADInstruments, NSW, Australia).

#### Nitrite Concentration

After the functional parameter measurements, all rats were deeply anesthetized by an overload of xylazine hydrochloride and ketamine hydrochloride (Anasedan^®^, 20 mg/kg and Dopalen^®^, 160 mg/kg, i.v., respectively, Ceva Sante Animale, Paulínea, SP, Brazil) and euthanized by decapitation. Blood samples were collected in heparinized vacutainers immediately after the euthanasia and centrifuged at 4,000 rpm for 5 min for analysis of nitrite concentration as previously published (Jacomini et al., 2017). In summary, proteins were quantified using automated biochemistry equipment (model A-15, Biosystems S/A, Barcelona, Spain) to normalize the calculation of nitrite concentration. Nitrites (NO_2_^–^), metabolites of NO, were determined in plasma using Griess reagent in which a chromophore with a strong absorbance at 540 nm is formed by the reaction of nitrite with a mixture of naphthyl ethylenediamine (0.1%) and sulfanilamide (1%). Samples were analyzed in duplicate, and plasma results are expressed as nmol/mg of protein.

### Proteomic Analysis

#### Protein Extraction

After euthanasia, the thoracic aorta was excised, cleaned, and homogenized in liquid nitrogen to prevent protein degradation. For the extraction, a total of 50 mg of tissue was homogenized in 500 μl of lysis buffer (7 M urea, 2 M thiourea, and 40 mM dithiothreitol (DTT), all diluted in 50 mM of AMBIC solution) for 2 h in the refrigerator, shaking all the time and, at the end, centrifuged at 20,817 *g* for 30 min at 4°C, followed by the collection of the supernatant. Total protein was quantified using the Quick Start^TM^ Bradford Protein Assay kit (Bio-Rad, Hercules, CA, United States), in duplicate, as described in the literature (Bradford, 1976).

#### Proteomic Analysis of the Aorta

The proteomic analysis was performed as previously described (Dionizio et al., 2018, 2020). A pool sample of aorta from two rats was performed and the proteomic analysis was done in biological triplicates. They were subdivided into 50-μl aliquots containing 50 μg of proteins (1 μg/μl) and 25 μl of a 0.2% RapiGest SF solution (Waters, Milford Massachusetts, United States) was then added, followed by agitation and 10 μl of 50 mM AMBIC was added. The samples were incubated at 37°C for 30 min. After this period, samples were reduced using 2.5 μl of 100 mM DTT (Merck KGaA, Darmstadt, Germany), incubated at 37°C for 60 min, alkylated with 2.5 μl of 300 mM iodoacetamide (IAA, Sigma-Aldrich, Darmstadt, Germany), agitated, and incubated in the dark at room temperature for 30 min. The samples were digested with the addition of 100 ng of trypsin solution (Thermo Scientific, Santa Clara, United States) in 50 mM AMBIC at 37°C overnight. After digestion, 10 μl of 5% trifluoroacetic acid (TFA) was added, agitated, and incubated at 37°C for 90 min. Subsequently, samples were centrifuged at 20,817 *g* at 6°C, for 30 min. The supernatants were purified and desalinated using a Pierce C18 Spin column (Thermo Scientific, Santa Clara, United States). The supernatant was resuspended in 108 μl 3% acetonitrile, 0.1% formic acid, and 12 μl of standard enolase. Peptide identification was performed on a nanoAcquity UPLC-Xevo QTof MS system (Waters, Manchester, United Kingdom) as previously described (Lima Leite et al., 2014). Protein identification was obtained using ProteinLynx Global Server (PLGS) version 3.0, using the ion-counting algorithm incorporated into the software. The data obtained were searched in the database of the species *Rattus norvegicus* (UniProtKB/Swiss-Prot). The protein profile was obtained using the CYTOSCAPE^®^ software v.3.7.0 (Java^®^ 1.8.0_162) and the plugins ClusterMarker and ClueGO. All proteins identified by the mass spectrometer were inserted into the software, using their access number, and can also be seen in the UniProt database, free of charge and available on the virtual platform (UniProt Consortium, 2019).

After confirming the proteins in the UniProt accession database, the first network was created. Then, it was necessary to make a filter with the taxonomy used in this study (*Rattus norvegicus*; 10,116). Within this classification, proteins were separated with a ratio value greater than one for those found to be upregulated, or a ratio less than one for those downregulated. Different numbers were assigned to identify the proteins specific to each group in the comparison.

Then, in CYTOSCAPE^®^ itself, it was necessary to select other subclassifications from the list to form networks with greater specificity and the possible protein comparisons that interacted with those identified by the mass spectrometer. CYTOSCAPE^®^ also has other interesting features such as plug-ins Clustermarker and ClueGo^®^, which allowed us to classify the proteins identified by the mass spectrometer according to their characteristics: biological process, relationship with the cellular component, immune system process, molecular function, KEGG (pathways involving genes and genome), REACTOME (biological pathways in humans), and WikiPathways (general biological pathways).

The proteins were analyzed and aggregated by the term that had the most meaning to describe them. In this way, genes, proteins, and mRNA can be connected and integrated within a subnetwork created by *Cytoscape*^®^ *software of the plug-in ClusterMark*^®^, which allows us to seek interrelationships to better investigate and to provide new potential associations, which can be created using the layout offered through ClueGo^®^.

### Statistical Analysis

All values are presented as mean ± standard error of the mean (SEM). For the samples with normal distribution, one-way analysis of variance (ANOVA) was used. Appropriate adjustments were made by Sigma Stat software for abnormal distribution samples. Two-way RM ANOVA was used for the longitudinal data of SBP. Pearson test was used for the correlation between functional and biochemical parameters. Tukey or Bonferroni *post hoc* tests were used when necessary. For the proteomic analysis, the comparison between groups was obtained using the PLGS software, employing Monte Carlo algorithm, considering *p* < 0.05 for the downregulated proteins and 1 – *p* > 0.95 for the upregulated proteins.

## Results

### Functional and Biochemical Analyses

At the beginning of the protocol, all groups presented similar BW. At the end of the experimental protocol, SHR groups, regardless of perindopril treatment, presented lower values of BW (415 ± 11; 305 ± 9, and 313 ± 16 g, for Wistar, SHR_C_, and SHR_P_, respectively, *p* < 0.0001).

**FIGURE 1:**
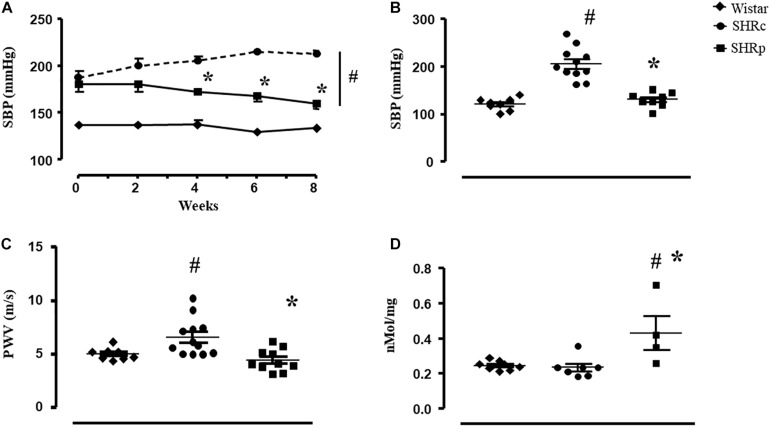
**(A)** Values of systolic blood pressure (SBP, mmHg, and tail-cuff technique) measured every other week in all groups: Wistar (*n* = 10), SHR_C_ (SHR_C_, *n* = 7), and SHR_P_ (SHR_P_, *n* = 5). **(B)** Values of systolic blood pressure (SBP, mmHg, and direct technique) measured at the end of the experimental protocol in all groups: Wistar (*n* = 8), SHR_C_ (SHR_C_, *n* = 11), and SHR_P_ (SHR_P_, *n* = 8). **(C)** Values of pulse wave velocity (PWV, m/s) measured at the end of the experimental protocol in all groups: Wistar (*n* = 8), SHR_C_ (SHR_C_, *n* = 12), and SHR_P_ (SHR_P_, *n* = 10). **(D)** Values of plasma nitrite concentration (nmol/mg) in all groups: Wistar (*n* = 8), SHR_C_ (SHR_C_, *n* = 12), and SHR_P_ (SHR_P_, *n* = 10). Significance: # vs Wistar and * vs control, *p* < 0.05.

We performed a tail-cuff pressure measurement at the beginning and during the experimental protocol to observe a time-course change of pressure during the protocol (Figure 1A). As shown, when perindopril started to be administered, both SHR groups presented higher SBP (tail-cuff) compared with Wistar rats (187 ± 7, 180 ± 8, and 137 ± 3 mmHg for SHR_C_, SHR_P_, and Wistar, respectively). From week 4 up to week 8, SHR_P_ presented lower SBP compared with SHR_C_ (*p* < 0.001). Perindopril treatment reduced the SBP of SHR up to 160 ± 4 mmHg (*p* = 0.02, vs beginning) while SHR_C_ maintained its higher values (213 ± 4 mmHg). During all 8 weeks, SBP of both groups of SHR was higher than that of Wistar rats. Figure 1B shows the values of direct BP measurement at the end of the experimental protocol. SHR_C_ presented higher values of SBP compared with Wistar rats (+70%) and treated SHR presented lower values of SBP compared with SHR_C_ (121 ± 10, 206 ± 10, and 131 ± 6 mmHg for Wistar, SHR_C_, and SHR_P_, respectively, *p* < 0.05).

Pulse wave velocity of the SHR_C_ group was higher than that of the Wistar group (+32%), and perindopril treatment attenuated this increase (5.0 ± 0.2, 6.5 ± 0.5, and 4.4 ± 0.3 m/s for Wistar, SHR_C_, and SHR_P_, respectively, *p* < 0.05), as shown in Figure 1C. Note that the PWV value of SHR_P_ was similar to that of the Wistar group. Pearson correlation test revealed a positive correlation between PWV and SBP (*r* = 0.410, *p* = 0.037), considering all groups of rats.

Figure 1D illustrates that perindopril treatment induced an increase on plasma nitrite concentration in SHR, since SHR_P_ presented higher values of plasma nitrite compared with SHR_C_ and Wistar groups (*p* ≤ 0.007). In addition, Pearson correlation test found a negative correlation between plasma nitrite concentration and PWV (*r* = −0.511, *p* = 0.034).

### Proteomic Analysis

As for the comparisons between the SHR_C_ × WISTAR groups, a total of 228 proteins were identified (Supplementary Table 1). From that, 86 proteins were uniquely identified in each group, 26 of which were related to the SHR_C_ group and 60 were related to the Wistar group. We obtained 142 of them with difference in expression, but only 42 reached significant statistical differences (Supplementary Table 1). Among them, 21 were upregulated and 21 downregulated in the first group of the comparison.

For the comparisons between SHR_P_ × SHR_C_, a total of 260 proteins were identified (Supplementary Table 2). Among them, a total of 122 were uniquely identified, 101 for SHR_P_ and 21 for SHR_C_. From the total of identified proteins, 138 showed differences in expression, but only 75 reached significant statistical difference (Supplementary Table 2). Among them, 73 were upregulated and two were downregulated as an effect of perindopril treatment.

**FIGURE 2:**
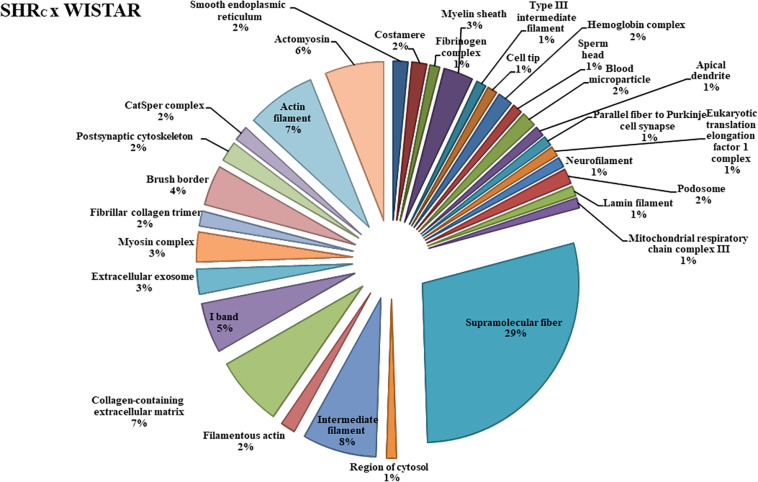
The protein distributions identified with the expression in the SHR_C_ × WISTAR comparison group. The categories are presented and based on the gene ontology according to the cellular component in which they participate, provided by the Cytoscape^®^ software v.3.7.0. Only significant terms were used, and the distribution was made according to the percentage of genes associated by category. The protein access numbers were made available by UNIPROT, while the gene ontology was analyzed by the Cytoscape^®^ software of the plug-in Cluego (Bindea et al., 2013).

**FIGURE 3:**
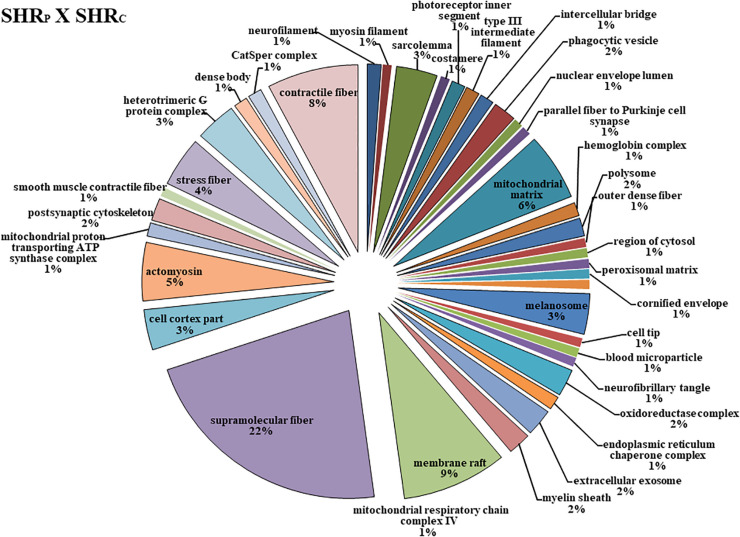
The protein distributions identified with the expression in the SHR_P_ × SHR_C_ comparison group. The categories are presented and based on the gene ontology according to the cellular component in which they participate, provided by the Cytoscape^®^ software v.3.7.0. Only significant terms were used, and the distribution was made according to the percentage of genes associated by category. The protein access numbers were made available by UNIPROT, while the gene ontology was analyzed by the Cytoscape^®^ software of the plug-in Cluego (Bindea et al., 2013).

The functional classification according to the cellular component is illustrated in Figure 2, for the SHR_C_ × WISTAR comparisons, and in Figure 3, for SHR_P_ × SHR_C_ comparisons. As shown in Figure 2, **30** components were changed by hypertension. Among them, the six most modified were Supramolecular Fiber (29%), Intermediate Filament (7.65%), Collagen-Containing Extracellular Matrix (7.14%), Actin Filament (7.14%), Actomyosin (6%), and I Band (5%).

Figure 3 shows that perindopril treatment determined more changes in the cellular component, that is, 38 types of components. Among them, the six most affected were Supramolecular Fiber (22%), Membrane Raft (9%), Contractile Fiber (8%), Mitochondrial Matrix (8%), Actomyosin (5%), and Stress Fiber (4%).

**FIGURE 4:**
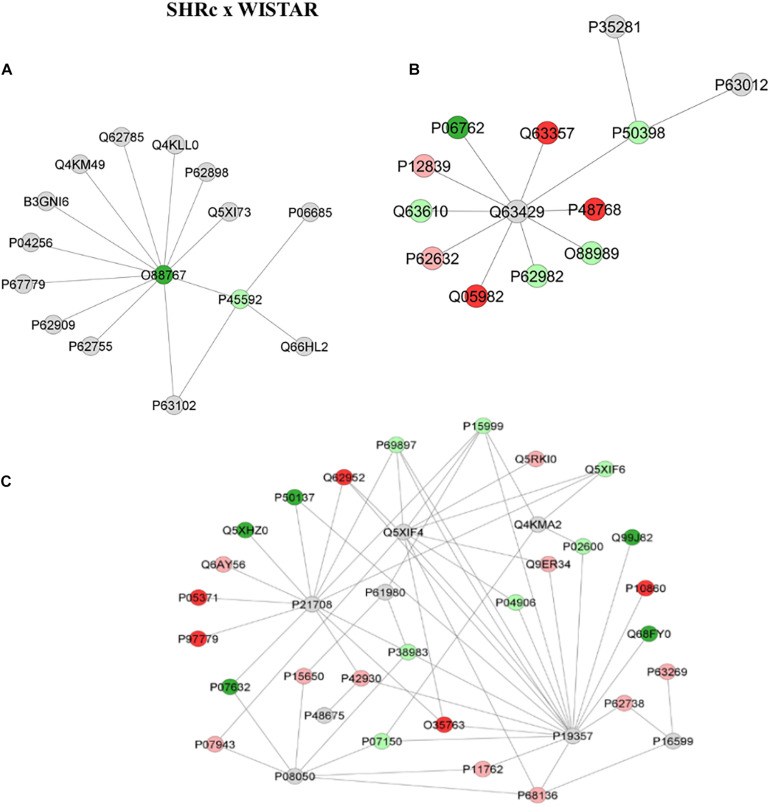
Proteins identified in the aorta of the SHR_C_ × Wistar group. The subnetworks created to demonstrate the interactions carried out by Cytoscape^®^ software of the plug-in ClusterMark. The color indicates the differential expression of the respective named protein with its access code; dark green denotes proteins belonging to the first group of the comparison, red indicates those belonging to the second group of the comparison, light pink represents downregulated proteins, light green denotes upregulated proteins, and gray indicates the interacting proteins. In **(A)**, light green denotes upregulated protein *cofilin-1* (P45592), dark green indicates protein *Parkinson disease protein 7 homolog* (O88767), and gray represents the interacting proteins *Sodium/potassium-transporting ATPase subunit alpha-1* (P06685), 14-3-3 protein zeta/delta (P63102), *Src substrate cortactin* (Q66HL2), *40S ribosomal protein S6* (P62755), *40S ribosomal protein S3* (P62909), *Prohibitin* (P67779), Heterogeneous nuclear ribonucleoprotein A1 (P04256), Septin-11 (B3GNI6), *Tyrosine–tRNA ligase cytoplasmic* (Q4KM49), *28 kDa heat- and acid-stable phosphoprotein* (Q62785), *Transcription elongation factor A protein 1* (Q4KLL0), *Cytochrome c somatic* (P62898), and *Rho GDP-dissociation inhibitor 1* (Q5XI73). In **(B)**, light green indicates upregulated protein *Rab GDP dissociation inhibitor alpha* (P50398) interacting with two gray proteins *Ras-related protein Rab-10* (P35281) and *Ras-related protein Rab-3^*a*^* (P63012); the other gray protein, *Polyubiquitin-C* (Q63429), is interacting with other proteins; dark green denotes the first group of the comparison *Heme oxygenase 1* (P06762), while the other three red ones represent those belonging to the second group of the comparison, *Unconventional myosin-Id* (Q63357), *Sodium/calcium exchanger 2* (P48768), *Nucleoside diphosphate kinase A* (Q05928), *Tropomyosin alpha-3 chain* (Q63610), *Ubiquitin-40S ribosomal protein S27a* (P62982), *Malate dehydrogenase*, and *cytoplasmic* (O88989); light green denotes upregulated proteins while light pink indicates downregulated proteins *Neurofilament medium polypeptide* (P12839) and *Elongation factor 1-alpha 2* (P62632). In **(C)**, gray denotes interaction proteins *Mitogen-activated protein kinase 3* (P21708), *Solute carrier family 2, facilitated glucose transporter member 4* (P19357), *UV excision repair protein RAD23 homolog B* (Q4KMA2), *Heterogeneous nuclear ribonucleoprotein K* (P61980), *Small ubiquitin-related modifier 3* (Q5XIF4), *Desmin* (P48675), *Gap junction alpha-1 protein* (P08050), and *Tumor necrosis factor* (P16599); light pink represents downregulated proteins *Long-chain specific acyl-CoA dehydrogenase, mitochondrial* (P15650), *Heat shock protein beta-1* (P42930), *Aldo-keto reductase family 1 member B1* (P07943), *Galectin-1* (P11762), *Actin, alpha skeletal muscle* (P68136), *Actin, aortic smooth muscle* (P62738), *Actin, gamma-enteric smooth muscle* (P63269), *Aconitate hydratase, mitochondrial* (Q9ER34), *Tubulin alpha-8 chain* (Q6AY56), *WD repeat-containing protein 1* (Q5RKI0), and *40S ribosomal protein AS* (P38983). *Annexin A1* (P07150), *Glutathione S-transferase P* (P04906), *Myosin light chain 1/3, skeletal muscle isoform* (P02600), *Tubulin alpha-4A chain* (Q5XIF6), *Tubulin beta-5 chain* (P69897), *ATP synthase subunit alpha, mitochondrial* (P15999) are in light green, denoting upregulated proteins; red ones are those belonging to the second group of the comparison *Moesin* (O35763), *Hyaluronan-mediated motility receptor* (P97779), *Clusterin* (P05371), *Glutamate dehydrogenase 1, mitochondrial* (P10860), and Dihydropyrimidinase-related protein 3 (Q62952); dark green ones belong to the first group of the comparison *Superoxide dismutase [Cu-Zn]* (P07632), *Cytochrome b-c1 complex subunit 1*, *mitochondrial* (Q68FY0), *Heat shock protein 75 kDa, mitochondrial* (Q5XHZ0), *Transketolase* (P50137), and *Integrin-linked protein kinase* (Q99J82).

**FIGURE 5:**
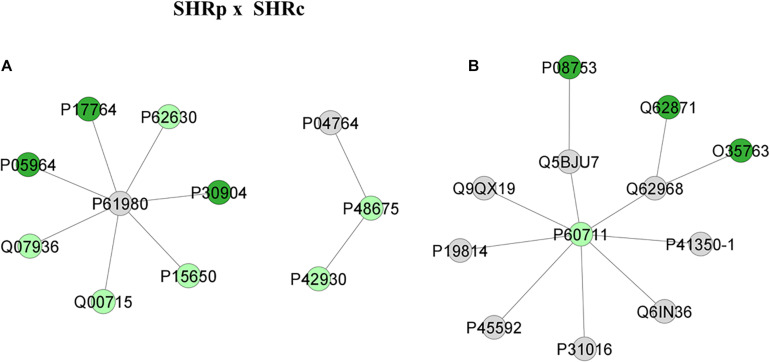
Proteins identified in the aorta of the SHR_C_ × Wistar group. The subnetworks created to demonstrate the interactions carried out by Cytoscape^®^ software of the plug-in ClusterMark. The color indicates the differential expression of the respective named protein with its access code; dark green denotes proteins belonging to the first group of the comparison; light green indicates upregulated proteins; and gray represents the interacting protein. In **(A)**, light green denotes Heat shock protein beta-1 (P42930), Desmin (P48675), *Long-chain specific acyl-CoA dehydrogenase, mitochondrial* (P15650), *Histone H2B type 1* (Q00715), *Annexin A2* (Q07936), and *Elongation factor 1-alpha 1* (P62630); dark green indicates proteins belonging to the first group, *Protein S100-A6* (P05964), *Acetyl-CoA acetyltransferase, mitochondrial* (P17764), and *Macrophage migration inhibitory factor* (P30904); gray represents the interacting proteins *Alpha-enolase* (P04764) and *Heterogeneous nuclear ribonucleoprotein K* (P61980). In **(B)** *Actin, cytoplasmic 1* (P60711) is the only protein upregulated, with networks *COFILIN-1* (P45592), *Sodium channel protein type 10 subunit alpha* (Q62968), *Trans-Golgi network integral membrane protein TGN38* (P19814), *Citron rho-interacting kinase* (Q9QX19), *Wiskott-Aldrich syndrome protein family member 1* (Q5BJU7), *Disks large homolog 4* (P31016), *WAS/WASL-interacting protein family member 1* (Q6IN36), and *Caveolin-1* (P41350-1); dark green indicates proteins belonging to the first group, *Moesin* (O35763), *Cytoplasmic dynein 1 intermediate chain 2* (Q62871), and *Guanine nucleotide-binding protein G(i) subunit alpha* (P08753).

Figure 4 shows the comparison network SHR_C_ × Wistar and Figure 5 shows the comparison between SHR_P_ × SHR_C_. These comparisons demonstrate changes in proteins during the process of hypertension and treatment with perindopril, respectively. Looking at the networks, each color represents a type of regulation: dark green denotes proteins belonging to the first group of the comparison only, red indicates those belonging to the second group of the comparison only, light pink represents downregulated proteins, light green denotes upregulated proteins, and gray represents the interacting proteins.

For the first comparison, between SHRc and Wistar, the network illustrated in Figure 4A shows a *Parkinson disease protein 7 homolog* (O88767), which was shown to belong to SHR_C_ interacting with several other proteins, but especially with an upregulated protein in the SHRc groups called *Cofilin-1* (P45592), which in turn interacts with *Sodium/potassium-transporting ATPase subunit alpha-1* (P06685), *14-3-3 protein zeta/delta* (P63102), and *Src substrate cortactin* (Q66HL2). Similarly, Figure 4B shows that *Rab GDP dissociation inhibitor alpha* (P50398), which is upregulated in SHR_C_ × Wistar, is interacting with three other upregulated proteins, *Malato dehydrogenase* (O88989), Ubiquitin (P62982), and Tropomyosin (O63610), through the interaction protein Polyubiquitin-C (Q63429). Also, *Rab GDP dissociation inhibitor alpha* (P50398) is interacting with *Ras-related protein Rab-10* (P35281) and *Ras-related protein Rab-3A* (P63012). In addition, Figure 4C illustrates another network with several modulated proteins in the aorta artery, within the SHRc × Wistar comparisons. It is possible to observe in this network that there were three main interaction proteins, *Mitogen-activated protein kinase 3* (P21708), *solute carrier muscle family-2 glucose transporter* (P19357), and *UV excision Rad23 homolog* (Q4KMA2), that interacted with several proteins up- or downregulated in the aorta sample, after SHRc × Wistar comparisons, like *long-chain specific acyl-CoA dehydrogenase-*mitochondrial (P15650), *heat shock protein beta-1* (P42930), *40S ribosomal protein AS* (P38983), and *Desmin* (P48675).

Figure 5 illustrates the networks after perindopril treatment that is between SHR_P_ × SHR_C_ groups. The first network (Figure 5A) reveals that the upregulated protein *Heat shock protein beta-1* (P42930) interacts also with an upregulated protein, *Desmin* (P48675). This network also shows that *long-chain specific acyl-CoA dehydrogenase, mitochondrial* (P15650) interacts with several other upregulated proteins through the *heterogeneous nuclear ribonucleoprotein K* (P61980). On the other hand, Figure 5B illustrates that *Actin, cytoplasmic 1* (P60711), which was upregulated after perindopril treatment, interacts with several other proteins, but none of them were altered by treatment, like *sodium channel protein type 10 subunit alpha* (Q62968), *moesin* (O35763), *cytoplasmic dynein 1 intermediate chain 2* (Q62871), and *cofilin-1* (P45592).

## Discussion

The main results observed in the present study were that perindopril treatment significantly reduced blood pressure and PWV in SHR. Also, plasma nitrite concentration was negatively correlated with PWV. The proteomic approach identified some differentially expressed proteins induced by hypertension and perindopril treatment, which may contribute to identify possible targets for the management of arterial stiffness.

Even though it is not clear whether arterial stiffness precedes hypertension or it is a consequence (Kaess et al., 2012; Mitchell, 2014; Celik et al., 2017; Rode et al., 2020), several studies have shown a significant correlation between PWV and BP (Laurent et al., 2009; Phillips et al., 2015; Steppan et al., 2020), including the present study, and the growing incidence of cardiovascular events in patients with high PWV values is eminent (Niiranen et al., 2017; Nilsson Wadstrom et al., 2019; Scuteri et al., 2020). Therefore, it is important to determine the mechanisms involved in vascular stiffening.

It is well known that angiotensin II (Ang II) plays a central role in hypertension due to its potent contractile action, and drugs that inhibit Ang II signaling are widely used to treat hypertension (Schmidt-Ott et al., 2000; Marque et al., 2002; Varagic et al., 2010; Gonzalez et al., 2018). While there are several classes of therapeutic agents to control pressure (Chobanian, 2017; Cuspidi et al., 2018; Unger et al., 2020), ACE inhibitors are highly recommended because of their cardio- and vascular protective effects (Ferrari et al., 2005; Janic et al., 2014; Chobanian, 2017). This study confirmed the participation of RAS in hypertension, as shown elsewhere (Marque et al., 2002; Safar, 2010; Hong et al., 2013; Natalin et al., 2016; Mancini et al., 2017; Steppan et al., 2020), since 8 weeks of perindopril treatment significantly reduced BP (−11%), as shown by the time course of SBP. We did not measure PWV at the beginning of the treatment, but since we have shown that PWV positively correlates with BP in SHR (Fabricio et al., 2020), we may assume that PWV was also high at the beginning of the protocol. Because RAS has also been implicated in vessel remodeling and aortic stiffening, ACE inhibitors play a crucial role in the restoration of the balance between plasma (and tissue) angiotensin II and bradykinin levels (Ferrari et al., 2005), which improves arteriolar structure, independent of their ability to reduce BP (Mancini et al., 2017). Ong et al. (2011) demonstrated in their meta-analysis that antihypertensive treatment can improve aortic stiffness beyond BP reduction in essential hypertensive patients and that the decrease in arterial stiffness was less under calcium antagonist treatment than under ACEi in a short-term trial, whereas all classes were equivalent in long-term trials.

Several clinical trials show interesting results, even though they are still inconclusive, but it is already known that other classes of anti-hypertensive drugs like diuretics and beta-blockers have few effects on PWV (Ong et al., 2011; Janic et al., 2014). Therefore, we chose to use perindopril, which has been shown to be a promising anti-hypertensive drug, capable to control/reverse artery stiffening, mainly because it causes changes on the mechanisms responsible to increase stiffness and it can be independent of BP reduction (Mahmud and Feely, 2002; Nakamura et al., 2005). In addition, perindopril is a prodrug ester that has a strong affinity for ACE and it inhibits 50% of ACE activity at a lower concentration than enalapril (Louis et al., 1992). Likewise, it was shown that lisinopril had about one-tenth the potency of perindopril with respect to its effects on plasma angiotensin peptide levels (Campbell et al., 1994). In addition, perindopril has high lipid solubility, it crosses the blood–brain barrier, and, because of that, it decreases brain ACE activity by 50%, different from enalapril and imidapril. For review, see Perini et al. (2020), which is a recent review that shows the pharmacological data of several ACEi. Also, it has been shown that perindopril reduces oxidative stress, which is another mechanism responsible to increase arterial stiffness (Ulu et al., 2014). Although there are several clinical trials investigating and demonstrating the effectiveness of perindopril, regular use in the clinical management of hypertension is not common yet (specially in public health units), mainly because of its elevated costs, when compared with other ACEi.

At the end of the experimental protocol, this study revealed that PWV and SBP were similar between Wistar and perindopril-treated SHR, but different from untreated SHR. We cannot avoid the possibility that PWV in SHR-treated rats could also be due to the hypotensive effect of perindopril. The use of another antihypertensive drug could help to solve this dilemma; however, it has been shown that clonidine as well as calcium blocker diltiazem reduced AP in parallel with a reduction of β-stiffness index and PWV (Vayssettes-Courchay et al., 2011; Lindesay et al., 2016, 2018), at least in young SHR. So, it seems that there is a lack of hypertension-independent arterial stiffening in young SHR, as suggested by Lindesay et al. (2016). On the other hand, it is important to note that the decrease on BP *per se* does not always reduce stiffness; for instance, Lindesay et al. (2016) demonstrated that when hypertension-induced vessel remodeling is already present, like in aged hypertensive rat, vessel distensibility remains, even after a pharmacological reduction of pressure (clonidine administration). In agreement, Mahmud and Feely (2002) have shown that PWV reduces independent of BP in hypertensive humans treated with Valsartan (AT1 antagonist) and captopril (ACEi). Recently, Steppan et al. (2020) showed that the restoration of normal BP in hypertensive mice (recovery period after stopping Ang II infusion) results in a partial recovery of overall *in vivo* stiffness (PWV). These authors suggest that restoration of BP improves the viscoelastic nature of blood vessels and partially recover the matrix mechanics, but the stiffness, which is irreversible, results from endothelial dysfunction and molecular changes to the vascular matrix, which contribute to VSMC dysregulation and are the major contributor to the overall *in vivo* vascular stiffness in essential hypertension. Therefore, further studies are still necessary to better understand the effects of perindopril treatment on PWV of SHR.

In addition, Ang II is a powerful mitogen that causes structural alterations in the vessel wall by stimulating the hypertrophy and hyperplasia of VSMCs, causing increases in the intralamellar distance on the vessel wall, increase of collagen, reduction of the elastin, and stiffness of intact and decellularized segments, among others (Wagenseil and Mecham, 2012; Mancini et al., 2017; Steppan et al., 2020). However, how exactly Ang II may modulate PWV is not completely known. Actually, there are several components that contribute to the arterial stiffening, like extracellular matrix (ECM) proteins that support the mechanical load, such as collagen and elastin (Lacolley et al., 2017). Lacerda et al. (2015) have suggested that PWV may be correlated with some components of the ECM in the vessel wall, like metalloproteinase biomarkers (MMP-9 and MMP-2) and collagen in patients with hypertensive heart disease; however, these biomarkers were evaluated in blood plasma. In addition, alterations of VSMCs, which regulate actomyosin interactions for contraction and cell–ECM interactions and depend on the architecture of cytoskeletal proteins and focal adhesion, are also contributing to regulate arterial stiffness; see Lacolley and colleagues for review (Lacolley et al., 2017). In agreement, phosphorylation or dephosphorylation of contractile proteins in VSMCs contributes to determine the dynamic modifications of the vessel diameter (Touyz et al., 2018). In fact, functional, structural, and biochemical alterations in the vessel wall have been investigated in different types of hypertension, but the molecular mechanisms involved remain unclear and most of these studies were performed *in vitro.* For this reason, in this study, we performed a proteomic analysis of the aorta tissue, excised from normotensive and hypertensive rats, treated or not with perindopril and compared SHR_C_ × Wistar to comprehend the effects of hypertension and SHR_P_ × SHR_C_ to identify the differentially expressed proteins after 8 weeks of perindopril treatment. As far as we know, this is the first work that aimed to identify the proteins related to vascular stiffening in aorta of SHR treated with perindopril.

Only few studies have analyzed the protein expression profile of the aorta during hypertension (Lee et al., 2004, 2006, 2009; Moriki et al., 2004; Bian et al., 2008; Feng et al., 2015; Lyck Hansen et al., 2015), and the results are fairly different. In the present study, when the functional classification according to the cellular component was performed, 30 different components were changed by hypertension (SHR_C_ × Wistar), and most of them were related to the mechanical integration of the various components of the cytoskeleton and responsible for physical support for cellular constituents, as shown in Figure 2. From 42 differentially expressed proteins in the aortic wall from SHR_C_ × Wistar rats (see Supplementary Table 1), 21 were upregulated and most of them were associated with cytoskeleton organization, stabilization of the aorta, and apoptosis like *Cofilin-1, Tubulin* β*-5 chain*, and *Tropomyosin alpha-3 chain*, among others (see Supplementary Table 1). In agreement, Lyck Hansen et al. (2015) have shown that several proteins related to smooth muscle cell function and organization of actin cytoskeleton, like *tubulin* β*-2A*, *tropomyosin* α*-4*, and α*-actinin 4*, among others, were significantly upregulated in patients with high PWV compared with those patients with normal PWV. In particular, these authors (Lyck Hansen et al., 2015) found that *Tropomyosin* α*-4* chain was a significant predictor of PWV. In the present study, *Tropomyosin* α*-4 chain* was expressed in SHR_C_’s aorta but did not reach statistical significance; however, *Tropomyosin* α*-3 chain* was upregulated. Using a two-dimensional electrophoresis system (2-DE), Bian et al. (2008) performed a proteomic analysis of the aorta from SHR and found that only two upregulated proteins were related to vessel stiffness: *GDP dissociation inhibitor protein* (*RhoGDIa*) and *Non-muscle myosin alkali light chain*.

It has been shown that Ang II levels in VSMC of hypertensive rats are higher than those in normotensive ones (Moriki et al., 2004) and Ang II is one of the main activators of the small GTPase family member (Moriki et al., 2004; Guan et al., 2013) and its downstream effector *Rho-associated protein kinase* (ROCK) (Zhou et al., 2017). In turn, activation on the RhoA/Rho-kinase pathway reduces the activity of myosin light-chain phosphatase (MLCP) through phosphorylation of its myosin targeting subunit (MYPT1) (Brozovich et al., 2016). This process sustains vasoconstriction, since myosin light chain (MLC) is not dephosphorylated by MLCP. In agreement, Zhou et al. (2017) demonstrated that VMSC from SHR’s aorta presents high activity of ROCK and high MYPT1 protein level. Similarly, Han et al. (2008) have shown that aorta of SHR had higher expression of myosin light chain kinase (MLCK) and myosin light chain phosphorylation (MLC-P), which in turn induces contraction (Guan et al., 2013).

In addition, ROCK is also an activator of LIM kinase-LIMK (Lacolley et al., 2017), an enzyme that phosphorylates and inactivates *Cofilin-1* (Morales-Quinones et al., 2020). Since the main activity of Cofilin-1 is to sever F-actin cytoskeletal stress fibers, inactivation of *Cofilin-1* reduces actin depolymerization (Lacolley et al., 2017; Sousa-Squiavinato et al., 2019) and induces arterial stiffening (Lam et al., 2007; Williams et al., 2019; Morales-Quinones et al., 2020). Although PWV of SHR was higher than that of Wistar, the present study showed that *Cofilin-1* was upregulated in stiffened aorta from SHR_C_ compared with Wistar rats. We may speculate that this increased *cofilin-1* expression could be a compensatory mechanism against aortic stiffening in SHRc, induced by high activity of the RhoA/Rho-kinase pathway, observed in hypertension (Moriki et al., 2004; Ying et al., 2004; Wynne et al., 2009; Walsh, 2011; Zhou et al., 2017). Furthermore, this *cofilin-1* could be dephosphorylated, as demonstrated by Lee et al. (2006). These authors have shown that *Cofilin-1* protein level was upregulated by hydrogen peroxide in rat aortic smooth muscle, but further analysis revealed that this cofilin-1 was dephosphorylated, which may lead to an inhibition of actin polymerization. Due to the nature of the proteomic analysis performed in the present study, this confirmation could not be performed, and future studies are necessary to confirm the activation state of *cofilin-1* in SHR’s aorta.

It is well known that reactive oxygen species (ROS) decreases nitric oxide (NO) bioavailability and induces hypertension and arterial stiffness (Landmesser et al., 2003; Bellien et al., 2010; Eleuterio-Silva et al., 2013; Roque et al., 2013; Wu et al., 2014). In agreement, using a 2D gel electrophoresis, Lee et al. (2009) identified seven proteins in the aorta artery that were differentially expressed between SHR and Wistar rats, including downregulation of the dihydropteridine reductase (DHPR), which is associated with the regeneration of tetra-hydrobiopterin (BH_4_) (Bendall et al., 2014). It has been shown that decreases in BH_4_ increases the generation of superoxide anion, which reduces NO bioavailability (Bellien et al., 2010). Reduction of NO availability has also been found after RhoA/ROCK pathway activation, which negatively regulates eNOS phosphorylation and eNOS expression (Ming et al., 2002).

In our study, we did not observe altered expression of DHPR or BH4 in aorta of SHR, but if we consider that hypertension and arterial stiffness are associated with reduction of NO bioavailability (induced by RhoA/Rho-kinase pathway activation or increases of ROS), we may hypothesize that increases in NO, due to perindopril treatment (indicated by the plasma nitrite concentration), could be involved in the PWV reduction observed in the present study in treated SHR (SHR_P_). Actually, the present study revealed that perindopril increased the nitrite concentration by 83% in SHR, and it was negatively correlated with PWV. In agreement with our results, other studies have shown that perindopril treatment increases plasma concentrations of nitrite/nitrate, indirectly indicating plasma NO contents (Kedziora-Kornatowska et al., 2006; Ceconi et al., 2007). Ceconi et al. (2007) have also observed an increase on eNOS protein expression and activity in coronary artery disease patients treated with perindopril. It has been shown that NO may relieve vascular stiffness by inactivation of RhoA/Rho-kinase pathway through a cGMP-dependent protein kinase activation (Krepinsky et al., 2003; Sauzeau et al., 2003). In fact, NO has been considered the most powerful physiological endothelial relaxing factor that negatively regulates *RhoA/Rho*-kinase activation in the vasculature. For review, see Nunes and Webb (2020).

In addition, we have identified an upregulation of *GDP dissociation inhibitor protein* (*GDIs*) in aorta of perindopril-treated SHR (SHR_P_), which is an internal regulator of *RhoA* activation. Actually, the activity of RhoA is normally controlled by three regulatory proteins, such as *guanine nucleotide exchange factors* (*GEFs*), *GTPase-activating proteins* (*GAPs*), and *GDP dissociation inhibitors* (*GDIs*) (Guan et al., 2013), the last one being an inhibitory protein, which is involved in the suppression of the transformation between Rho-GDP and Rho-GTP forms (Guan et al., 2013) and may contribute to a decrease in the *RhoA/ROCK/LIMK/Cofilin-1* pathway. Recently, Morales-Quinones et al. (2020) have shown that inhibition of LIMK reduces p-Cofilin/Cofilin and reduces arterial stiffness, which suggests the involvement of RhoA/ROCK/LIMK/Cofilin-1 on vascular stiffening. Similarly, losartan (an Ang II receptor type 1 antagonist) inhibits *RhoA/Rho-kinase* pathway activity in hypertensive rats (Moriki et al., 2004; Wynne et al., 2009). Likewise, inhibition of this *RhoA/Rho-kinase* pathway by Y-27632 (an inhibitor of ROCK) has been associated with reduction of BP (Seko et al., 2003; Moriki et al., 2004; Zhou et al., 2017) and vascular stiffness (Wehrwein et al., 2004). In agreement, Masumoto et al. (2001) have shown that Fasudil, an inhibitor of *Rho-kinase*, increased forearm blood flow in humans.

Moreover, the proteins *Long-chain specific acyl-CoA dehydrogenase mitochondrial* and *Heat shock protein beta-1*, interacting with *Desmin* in the network of SHR_P_ × SHR_C_ comparison, were downregulated in aorta of SHR_C_ and became upregulated after perindopril treatment. *Long-chain specific acyl-CoA dehydrogenase mitochondrial* is involved with the energy production and *Heat shock protein beta-1* is involved in protein folding. Interesting, Feng et al. (2015) identified the same proteins after physical exercise in aorta of SHR, which means that perindopril treatment may be contributing to restart the normal function of the vessel, as well as exercise training does.

In summary, the results of the present study revealed that treatment with perindopril reduced arterial pressure and PWV in SHR. In addition, the proteomic analysis in aorta suggested for the first time that *RhoA/Rho-kinase/LIMK/Cofilin-1* pathway may be inhibited by perindopril-induced upregulation of *GDIs* or increases in NO bioavailability in SHR. Therefore, we may propose that activation of *GDIs* or inhibition of *RhoA/Rho-kinase* pathway could be a possible strategy to treat arterial stiffness.

This study has some limitations. First, although we have shown a positive correlation between PWV and SBP, we cannot say that perindopril treatment improved PWV, since we did not measure PWV at the beginning of the experimental protocol; however, we may say that SHR-treated rats showed lower PWV compared with non-treated SHR. Second, since perindopril reduced pressure and PWV, we cannot avoid the possibility that PWV response was dependent of the BP, even though some studies have shown that sometimes arterial stiffness reduction may be independent of BP reduction; third, due to the limitation of the technique, PWV was measured in anesthetized rats. Finally, due to the nature of the proteomic analysis performed in the present study, we cannot confirm if the upregulation of the *cofilin-1* in SHR’s aorta is a compensatory mechanism or if this protein is dephosphorylated. Future studies, using more specific techniques, are necessary to further confirm the results observed in this study. Besides that, we do believe that this kind of study is important, mainly because the currently available studies using proteomics looking for a better management of hypertension and cardiovascular diseases are relatively small, not standardized, and difficult to compare with each other (Delles et al., 2018).

## Data Availability Statement

The original contributions presented in the study are included in the article/Supplementary Material, further inquiries can be directed to the corresponding author/s.

## Ethics Statement

The animal study was reviewed and approved by Committee for Ethical Use of Animals at School of Sciences, UNESP (#778/2017 vol.1).

## Author Contributions

SA and DM designed the study and wrote the manuscript. DM, AD, and AJ conducted the experiments, acquired the data, and analyzed the data. AZ supervised nitrite concentration experiments. SA and MB supervised the study. All authors contributed to the article and approved the submitted version.

## Conflict of Interest

The authors declare that the research was conducted in the absence of any commercial or financial relationships that could be construed as a potential conflict of interest. The handling editor declared a shared affiliation with several of the authors, AD and MB, at the time of review.

## Abbreviations

AngII: angiotensin II
BP: blood pressure
DHPR: dihydropteridine reductase
ECM: extracellular matrix
GDI: GDP dissociation inhibitor protein
GAPs: GTPase-activating proteins
GEFs: guanine nucleotide exchange factors
LIMK: LIM kinase
MMP: metalloproteinase
MLC: myosin light chain
MLCK: myosin light-chain kinase
MLC-P: myosin light-chain phosphorylation
MLCP: myosin light-chain phosphatase
MYPT1: myosin targeting subunit
NO: nitric oxide
NO_2_^–^: nitrite
PWV: pulse wave velocity
ROS: reactive oxygen species
RAS: renin–angiotensin system
SHR: spontaneously hypertensive rats
SBP: systolic blood pressure
BH4: tetra-hydrobiopterin
TT: transit time
VSMCs: vascular smooth muscle cells.
