# Supplementary material for: Identification of Aortic Proteins Involved in Arterial Stiffness in Spontaneously Hypertensive Rats Treated With Perindopril:A Proteomic Approach
Source: Front Physiol. 2021 Feb 10;12:624515. doi: 10.3389/fphys.2021.624515 (PMC7928294; doi:10.3389/fphys.2021.624515)
Supplement: Supplementary file 1 [file Table_1.DOCX]

**Table S1**. Proteins with expression significantly altered in the aorta of rats in the SHR_C_ X Wistar comparison.

| Accession Number | Protein Name | Score | Ratio  SHR_C_ / WISTAR |
| --- | --- | --- | --- |
| P63018 | Heat shock cognate 71 kDa protein | 721 | 3.78 |
| P04797 | Glyceraldehyde-3-phosphate dehydrogenase | 722 | 2.77 |
| P62260 | 14-3-3 protein epsilon | 183 | 2.59 |
| P15999 | ATP synthase subunit alpha_ mitochondrial | 511 | 2.25 |
| P47819 | Glial fibrillary acidic protein | 94 | 1.92 |
| P25113 | Phosphoglycerate mutase 1 | 190 | 1.88 |
| P02770 | Serum albumin | 3896 | 1.73 |
| O88989 | Malate dehydrogenase_ cytoplasmic | 142 | 1.72 |
| Q6IG12 | Keratin_ type II cytoskeletal 7 | 237 | 1.55 |
| P02600 | Myosin light chain 1/3_ skeletal muscle isoform | 201 | 1.51 |
| P62982 | Ubiquitin-40S ribosomal protein S27a | 335 | 1.45 |
| P20760 | Ig gamma-2A chain C region | 1932 | 1.45 |
| P85973 | Purine nucleoside phosphorylase | 395 | 1.42 |
| Q66HD0 | Endoplasmin | 29 | 1.39 |
| P04906 | Glutathione S-transferase P | 110 | 1.32 |
| Q5XIF6 | Tubulin alpha-4A chain | 336 | 1.31 |
| P07150 | Annexin A1 | 157 | 1.28 |
| P50398 | Rab GDP dissociation inhibitor alpha | 57 | 1.26 |
| Q63610 | Tropomyosin alpha-3 chain | 88 | 1.26 |
| Q6P9T8 | Tubulin beta-4B chain | 869 | 1.25 |
| P11598 | Protein disulfide-isomerase A3 | 70 | 1.25 |
| P62963 | Profilin-1 | 4776 | 1.23 |
| P08010 | Glutathione S-transferase Mu 2 | 1173 | 1.23 |
| P51886 | Lumican | 1174 | 1.21 |
| P63102 | 14-3-3 protein zeta/delta | 261 | 1.21 |
| P69897 | Tubulin beta-5 chain | 1244 | 1.20 |
| P58775 | Tropomyosin beta chain | 674 | 1.20 |
| P38983 | 40S ribosomal protein SA | 72 | 1.17 |
| P0DMW0 | Heat shock 70 kDa protein 1A | 580 | 1.17 |
| Q4FZU2 | Keratin_ type II cytoskeletal 6A | 94 | 1.17 |
| Q4V8H8 | EH domain-containing protein 2 | 75 | 1.17 |
| P85125 | Caveolae-associated protein 1 | 140 | 1.16 |
| Q07936 | Annexin A2 | 183 | 1.16 |
| Q6AYZ1 | Tubulin alpha-1C chain | 2075 | 1.16 |
| P68511 | 14-3-3 protein eta | 163 | 1.15 |
| Q5XI73 | Rho GDP-dissociation inhibitor 1 | 550 | 1.15 |
| P09495 | Tropomyosin alpha-4 chain | 88 | 1.15 |
| Q62812 | Myosin-9 | 126 | 1.15 |
| P0CG51 | Polyubiquitin-B | 335 | 1.15 |
| P02680 | Fibrinogen gamma chain | 110 | 1.14 |
| Q6IG05 | Keratin_ type II cytoskeletal 75 | 94 | 1.14 |
| P06866 | Haptoglobin | 102 | 1.13 |
| P24090 | Alpha-2-HS-glycoprotein | 172 | 1.13 |
| P06399 | Fibrinogen alpha chain | 89 | 1.13 |
| P05065 | Fructose-bisphosphate aldolase A | 156 | 1.12 |
| P85972 | Vinculin | 1691 | 1.12 |
| Q00715 | Histone H2B type 1 | 391 | 1.09 |
| P04692 | Tropomyosin alpha-1 chain | 666 | 1.09 |
| Q3KRE8 | Tubulin beta-2B chain | 1141 | 1.09 |
| P12346 | Serotransferrin | 288 | 1.09 |
| P45592 | Cofilin-1 | 751 | 1.08 |
| Q9QXQ0 | Alpha-actinin-4 | 299 | 1.08 |
| P70623 | Fatty acid-binding protein_ adipocyte | 328 | 1.08 |
| P62986 | Ubiquitin-60S ribosomal protein L40 | 335 | 1.07 |
| P11884 | Aldehyde dehydrogenase_ mitochondrial | 137 | 1.07 |
| P02454 | Collagen alpha-1(I) chain | 71 | 1.07 |
| P48675 | Desmin | 490 | 1.07 |
| Q7M0E3 | Destrin | 1860 | 1.06 |
| A6YP92 | Homeobox protein ARX | 105 | 1.06 |
| Q4QRB4 | Tubulin beta-3 chain | 680 | 1.05 |
| P14668 | Annexin A5 | 68 | 1.05 |
| Q10758 | Keratin_ type II cytoskeletal 8 | 237 | 1.05 |
| Q01129 | Decorin | 86 | 1.04 |
| Q9WVH8 | Fibulin-5 | 6538 | 1.04 |
| Q9Z1P2 | Alpha-actinin-1 | 356 | 1.04 |
| P02466 | Collagen alpha-2(I) chain | 148 | 1.04 |
| P48679 | Prelamin-A/C | 800 | 1.03 |
| P0DMW1 | Heat shock 70 kDa protein 1B | 580 | 1.03 |
| P15800 | Laminin subunit beta-2 | 71 | 1.02 |
| P11980 | Pyruvate kinase PKM | 87 | 1.01 |
| P01836 | Ig kappa chain C region_ A allele | 530 | 1.01 |
| P56574 | Isocitrate dehydrogenase [NADP]_ mitochondrial | 96 | 1.01 |
| P14480 | Fibrinogen beta chain | 258 | 0.99 |
| P62630 | Elongation factor 1-alpha 1 | 248 | 0.99 |
| P16409 | Myosin light chain 3 | 201 | 0.98 |
| Q08163 | Adenylyl cyclase-associated protein 1 | 513 | 0.98 |
| P31000 | Vimentin | 2129 | 0.97 |
| Q62736 | Non-muscle caldesmon | 207 | 0.97 |
| P55063 | Heat shock 70 kDa protein 1-like | 523 | 0.97 |
| P14659 | Heat shock-related 70 kDa protein 2 | 575 | 0.96 |
| P09117 | Fructose-bisphosphate aldolase C | 78 | 0.96 |
| P15429 | Beta-enolase | 76 | 0.96 |
| P06761 | Endoplasmic reticulum chaperone BiP | 355 | 0.96 |
| P85108 | Tubulin beta-2A chain | 1141 | 0.95 |
| P61983 | 14-3-3 protein gamma | 155 | 0.95 |
| P04937 | Fibronectin | 707 | 0.95 |
| P47942 | Dihydropyrimidinase-related protein 2 | 98 | 0.94 |
| P35213 | 14-3-3 protein beta/alpha | 155 | 0.94 |
| Q68FR8 | Tubulin alpha-3 chain | 383 | 0.94 |
| P01048 | T-kininogen 1 | 51 | 0.93 |
| Q6IG00 | Keratin_ type II cytoskeletal 4 | 114 | 0.93 |
| P34058 | Heat shock protein HSP 90-beta | 69 | 0.92 |
| P50399 | Rab GDP dissociation inhibitor beta | 100 | 0.92 |
| P10111 | Peptidyl-prolyl cis-trans isomerase A | 1492 | 0.92 |
| P47853 | Biglycan | 1012 | 0.92 |
| P47875 | Cysteine and glycine-rich protein 1 | 1823 | 0.92 |
| P11517 | Hemoglobin subunit beta-2 | 643 | 0.92 |
| P23565 | Alpha-internexin | 98 | 0.91 |
| P16617 | Phosphoglycerate kinase 1 | 58 | 0.91 |
| P18666 | Myosin regulatory light chain 12B | 766 | 0.91 |
| P70490 | Lactadherin | 1000 | 0.90 |
| P09006 | Serine protease inhibitor A3N | 258 | 0.90 |
| P48037 | Annexin A6 | 128 | 0.90 |
| P16636 | Protein-lysine 6-oxidase | 281 | 0.90 |
| Q64122 | Myosin regulatory light polypeptide 9 | 3264 | 0.90 |
| Q63429 | Polyubiquitin-C | 335 | 0.88 |
| P68370 | Tubulin alpha-1A chain | 2132 | 0.88 |
| P68255 | 14-3-3 protein theta | 155 | 0.88 |
| Q63598 | Plastin-3 | 85 | 0.87 |
| Q9ER34 | Aconitate hydratase_ mitochondrial | 71 | 0.87 |
| P01026 | Complement C3 | 90 | 0.85 |
| Q64119 | Myosin light polypeptide 6 | 14590 | 0.85 |
| P63259 | Actin_ cytoplasmic 2 | 28287 | 0.85 |
| P68136 | Actin_ alpha skeletal muscle | 32690 | 0.85 |
| Q9JLT0 | Myosin-10 | 73 | 0.84 |
| P10719 | ATP synthase subunit beta_ mitochondrial | 564 | 0.84 |
| P68035 | Actin_ alpha cardiac muscle 1 | 47537 | 0.84 |
| Q6P9V9 | Tubulin alpha-1B chain | 2106 | 0.83 |
| P20059 | Hemopexin | 158 | 0.83 |
| P62738 | Actin_ aortic smooth muscle | 48292 | 0.83 |
| P42930 | Heat shock protein beta-1 | 1538 | 0.82 |
| P15650 | Long-chain specific acyl-CoA dehydrogenase_ mitochondrial | 107 | 0.82 |
| Q6AY56 | Tubulin alpha-8 chain | 117 | 0.82 |
| P02091 | Hemoglobin subunit beta-1 | 1027 | 0.81 |
| P36201 | Cysteine-rich protein 2 | 173 | 0.80 |
| P01946 | Hemoglobin subunit alpha-1/2 | 6753 | 0.79 |
| Q68FP1 | Gelsolin | 140 | 0.78 |
| P04636 | Malate dehydrogenase_ mitochondrial | 110 | 0.78 |
| P12839 | Neurofilament medium polypeptide | 119 | 0.78 |
| P31232 | Transgelin | 5387 | 0.77 |
| P60711 | Actin_ cytoplasmic 1 | 28287 | 0.77 |
| P20761 | Ig gamma-2B chain C region | 226 | 0.76 |
| P20759 | Ig gamma-1 chain C region | 58 | 0.76 |
| P11762 | Galectin-1 | 1212 | 0.71 |
| Q6P6Q2 | Keratin_ type II cytoskeletal 5 | 110 | 0.69 |
| P21807 | Peripherin | 213 | 0.64 |
| P63269 | Actin_ gamma-enteric smooth muscle | 48292 | 0.64 |
| P13832 | Myosin regulatory light chain RLC-A | 766 | 0.59 |
| Q5RKI0 | WD repeat-containing protein 1 | 160 | 0.59 |
| P04764 | Alpha-enolase | 78 | 0.57 |
| P62632 | Elongation factor 1-alpha 2 | 102 | 0.45 |
| P07943 | Aldose reductase | 74 | 0.41 |
| Q6AXQ5 | 2'_5'-phosphodiesterase 12 | 45 | WISTAR* |
| Q78E60 | Aryl hydrocarbon receptor nuclear translocator 2 | 61 | WISTAR |
| Q03070 | Beta-chimaerin | 344 | WISTAR |
| Q3ZB98 | Breast carcinoma-amplified sequence 1 homolog | 39 | WISTAR |
| P0DP29 | Calmodulin-1 | 383 | WISTAR |
| P0DP30 | Calmodulin-2 | 350 | WISTAR |
| P0DP31 | Calmodulin-3 | 350 | WISTAR |
| Q08290 | Calponin-1 | 116 | WISTAR |
| O08556 | C-C chemokine receptor type 5 | 65 | WISTAR |
| O35112 | CD166 antigen | 139 | WISTAR |
| P11442 | Clathrin heavy chain 1 | 32 | WISTAR |
| P05371 | Clusterin | 75 | WISTAR |
| Q4KM47 | Cyclin-dependent kinase 10 | 271 | WISTAR |
| P20788 | Cytochrome b-c1 complex subunit Rieske_ mitochondrial | 67 | WISTAR |
| P13107 | Cytochrome P450 2B3 | 68 | WISTAR |
| Q62871 | Cytoplasmic dynein 1 intermediate chain 2 | 113 | WISTAR |
| Q62952 | Dihydropyrimidinase-related protein 3 | 93 | WISTAR |
| P51400 | Double-stranded RNA-specific editase 1 | 30 | WISTAR |
| Q3B8Q2 | Eukaryotic initiation factor 4A-III | 58 | WISTAR |
| A0JPM9 | Eukaryotic translation initiation factor 3 subunit J | 211 | WISTAR |
| P50609 | Fibromodulin | 180 | WISTAR |
| Q9WUH4 | Four and a half LIM domains protein 1 | 667 | WISTAR |
| P15431 | Gamma-aminobutyric acid receptor subunit beta-1 | 73 | WISTAR |
| P10860 | Glutamate dehydrogenase 1_ mitochondrial | 62 | WISTAR |
| Q9WTT6 | Guanine deaminase | 64 | WISTAR |
| P23899 | Hepatocyte nuclear factor 1-beta | 82 | WISTAR |
| Q99MK2 | Histone acetyltransferase KAT5 | 62 | WISTAR |
| Q00729 | Histone H2B type 1-A | 1870 | WISTAR |
| O35767 | Homeobox protein Nkx-2.5 | 81 | WISTAR |
| Q9ESM2 | Hyaluronan and proteoglycan link protein 2 | 40 | WISTAR |
| P97779 | Hyaluronan-mediated motility receptor | 34 | WISTAR |
| P20762 | Ig gamma-2C chain C region | 98 | WISTAR |
| P01835 | Ig kappa chain C region_ B allele | 4714 | WISTAR |
| Q9QYU4 | Ketimine reductase mu-crystallin | 158 | WISTAR |
| Q8R426 | Kv channel-interacting protein 1 | 126 | WISTAR |
| Q62733 | Lamina-associated polypeptide 2_ isoform beta | 58 | WISTAR |
| P70615 | Lamin-B1 | 74 | WISTAR |
| Q5XI07 | Lipoma-preferred partner homolog | 402 | WISTAR |
| Q5HZA4 | LysM and putative peptidoglycan-binding domain-containing protein 1 | 72 | WISTAR |
| Q4V8B3 | Mediator of RNA polymerase II transcription subunit 24 | 76 | WISTAR |
| O35763 | Moesin | 101 | WISTAR |
| A0A096MK47 | Muscular LMNA-interacting protein | 67 | WISTAR |
| P16884 | Neurofilament heavy polypeptide | 62 | WISTAR |
| Q05982 | Nucleoside diphosphate kinase A | 156 | WISTAR |
| P19804 | Nucleoside diphosphate kinase B | 156 | WISTAR |
| P52944 | PDZ and LIM domain protein 1 | 174 | WISTAR |
| Q66HS7 | PDZ and LIM domain protein 3 | 131 | WISTAR |
| Q62920 | PDZ and LIM domain protein 5 | 72 | WISTAR |
| Q9Z1Z9 | PDZ and LIM domain protein 7 | 143 | WISTAR |
| Q9QYU1 | Peroxisomal biogenesis factor 19 | 105 | WISTAR |
| Q9EQP5 | Prolargin | 148 | WISTAR |
| Q9ES87 | Prostasin | 225 | WISTAR |
| Q9QZQ5 | Protein NOV homolog | 231 | WISTAR |
| Q62796 | RalA-binding protein 1 | 46 | WISTAR |
| Q64604 | Receptor-type tyrosine-protein phosphatase F | 40 | WISTAR |
| Q91Y81 | Septin-2 | 98 | WISTAR |
| P48768 | Sodium/calcium exchanger 2 | 74 | WISTAR |
| P08932 | T-kininogen 2 | 47 | WISTAR |
| Q9EQT5 | Tubulointerstitial nephritis antigen-like | 132 | WISTAR |
| Q63357 | Unconventional myosin-Id | 61 | WISTAR |
| P13437 | 3-ketoacyl-CoA thiolase_ mitochondrial | 85 | SHR_C_* |
| P80299 | Bifunctional epoxide hydrolase 2 | 47 | SHR_C_ |
| Q4V8E4 | Cilia- and flagella-associated protein 36 | 83 | SHR_C_ |
| Q68FY0 | Cytochrome b-c1 complex subunit 1_ mitochondrial | 160 | SHR_C_ |
| O55096 | Dipeptidyl peptidase 3 | 45 | SHR_C_ |
| Q91XQ4 | DNA-directed RNA polymerase II subunit GRINL1A | 69 | SHR_C_ |
| Q62862 | Dual specificity mitogen-activated protein kinase kinase 5 | 67 | SHR_C_ |
| Q5EZ72 | Ectonucleotide pyrophosphatase/phosphodiesterase family member 7 | 42 | SHR_C_ |
| Q68FU3 | Electron transfer flavoprotein subunit beta | 536 | SHR_C_ |
| Q66H04 | F-box only protein 43 | 68 | SHR_C_ |
| P13255 | Glycine N-methyltransferase | 44 | SHR_C_ |
| Q5XHZ0 | Heat shock protein 75 kDa_ mitochondrial | 47 | SHR_C_ |
| P06762 | Heme oxygenase 1 | 60 | SHR_C_ |
| Q6IE24 | Inactive ubiquitin carboxyl-terminal hydrolase 54 | 36 | SHR_C_ |
| E9PU28 | Inosine-5'-monophosphate dehydrogenase 2 =1 | 79 | SHR_C_ |
| Q99J82 | Integrin-linked protein kinase | 75 | SHR_C_ |
| P18588 | Interferon-induced GTP-binding protein Mx1 | 61 | SHR_C_ |
| Q5U2U7 | mRNA cap guanine-N7 methyltransferase | 99 | SHR_C_ |
| A7E3N2 | Neutrophil cytosol factor 2 | 52 | SHR_C_ |
| P11506 | Plasma membrane calcium-transporting ATPase 2 | 71 | SHR_C_ |
| O88767 | Protein/nucleic acid deglycase DJ-1 | 229 | SHR_C_ |
| Q9JK11 | Reticulon-4 | 39 | SHR_C_ |
| P42346 | Serine/threonine-protein kinase mTOR | 52 | SHR_C_ |
| D3ZVU1 | SprT-like domain-containing protein Spartan | 72 | SHR_C_ |
| P07632 | Superoxide dismutase [Cu-Zn] | 125 | SHR_C_ |
| P50137 | Transketolase | 50 | SHR_C_ |

^a^Identification is based on proteins ID from UniProt protein database, reviewed only (<http://www.uniprot.org/>).

^b^Proteins with expression significantly altered are organized according to the ratio.

*Indicates unique proteins in alphabetical order.
