# Supplementary material for: Identification of Aortic Proteins Involved in Arterial Stiffness in Spontaneously Hypertensive Rats Treated With Perindopril:A Proteomic Approach
Source: Front Physiol. 2021 Feb 10;12:624515. doi: 10.3389/fphys.2021.624515 (PMC7928294; doi:10.3389/fphys.2021.624515)
Supplement: Supplementary file 2 [file Table_2.DOCX]

**Table S2**. Proteins with expression significantly altered in the aorta of rats in the SHR_P_ X SHR_C_ comparison.

| ^a^Accession Number | Protein Name | Score | ^b^Ratio  SHR_P_/SHR_C_ |
| --- | --- | --- | --- |
| Q6AY56 | Tubulin alpha-8 chain | 46 | 2.20 |
| P62632 | Elongation factor 1-alpha 2 | 51 | 2.03 |
| P02454 | Collagen alpha-1(I) chain | 47 | 1.88 |
| Q68FR8 | Tubulin alpha-3 chain | 111 | 1.84 |
| P50398 | Rab GDP dissociation inhibitor alpha | 112 | 1.79 |
| P11762 | Galectin-1 | 260 | 1.75 |
| A6YP92 | Homeobox protein ARX | 33 | 1.75 |
| Q5XIF6 | Tubulin alpha-4A chain | 107 | 1.73 |
| P47853 | Biglycan | 227 | 1.72 |
| Q62736 | Non-muscle caldesmon | 274 | 1.72 |
| Q10758 | Keratin_ type II cytoskeletal 8 | 193 | 1.70 |
| Q6P6Q2 | Keratin_ type II cytoskeletal 5 | 76 | 1.70 |
| Q6AYZ1 | Tubulin alpha-1C chain | 237 | 1.68 |
| Q9ER34 | Aconitate hydratase_ mitochondrial | 83 | 1.67 |
| Q6IG12 | Keratin_ type II cytoskeletal 7 | 193 | 1.67 |
| P68370 | Tubulin alpha-1A chain | 237 | 1.67 |
| Q6P9V9 | Tubulin alpha-1B chain | 237 | 1.67 |
| P34058 | Heat shock protein HSP 90-beta | 106 | 1.67 |
| P50399 | Rab GDP dissociation inhibitor beta | 52 | 1.65 |
| P36201 | Cysteine-rich protein 2 | 176 | 1.63 |
| P62630 | Elongation factor 1-alpha 1 | 298 | 1.62 |
| P70623 | Fatty acid-binding protein_ adipocyte | 436 | 1.60 |
| P08010 | Glutathione S-transferase Mu 2 | 217 | 1.58 |
| Q01129 | Decorin | 150 | 1.55 |
| P25113 | Phosphoglycerate mutase 1 | 276 | 1.55 |
| P16617 | Phosphoglycerate kinase 1 | 73 | 1.55 |
| P15999 | ATP synthase subunit alpha_ mitochondrial | 175 | 1.54 |
| P42930 | Heat shock protein beta-1 | 848 | 1.54 |
| Q4FZU2 | Keratin_ type II cytoskeletal 6A | 76 | 1.54 |
| Q62812 | Myosin-9 | 68 | 1.52 |
| P07150 | Annexin A1 | 94 | 1.52 |
| P85125 | Caveolae-associated protein 1 | 150 | 1.51 |
| P15650 | Long-chain specific acyl-CoA dehydrogenase_ mitochondrial | 93 | 1.51 |
| P11980 | Pyruvate kinase PKM | 40 | 1.51 |
| P09117 | Fructose-bisphosphate aldolase C | 79 | 1.51 |
| Q9JLT0 | Myosin-10 | 71 | 1.49 |
| P85108 | Tubulin beta-2A chain | 94 | 1.49 |
| Q3KRE8 | Tubulin beta-2B chain | 94 | 1.49 |
| P69897 | Tubulin beta-5 chain | 94 | 1.49 |
| Q6IG00 | Keratin_ type II cytoskeletal 4 | 52 | 1.49 |
| Q4QRB4 | Tubulin beta-3 chain | 94 | 1.48 |
| Q6P9T8 | Tubulin beta-4B chain | 467 | 1.48 |
| Q00715 | Histone H2B type 1 | 122 | 1.48 |
| P16636 | Protein-lysine 6-oxidase | 251 | 1.46 |
| P47875 | Cysteine and glycine-rich protein 1 | 1148 | 1.45 |
| Q4V8H8 | EH domain-containing protein 2 | 225 | 1.45 |
| Q6IG05 | Keratin_ type II cytoskeletal 75 | 76 | 1.45 |
| P47819 | Glial fibrillary acidic protein | 52 | 1.45 |
| P10111 | Peptidyl-prolyl cis-trans isomerase A | 1750 | 1.42 |
| P11598 | Protein disulfide-isomerase A3 | 100 | 1.42 |
| P31232 | Transgelin | 9127 | 1.40 |
| P04636 | Malate dehydrogenase_ mitochondrial | 346 | 1.40 |
| P50137 | Transketolase | 50 | 1.40 |
| P11506 | Plasma membrane calcium-transporting ATPase 2 | 71 | 1.40 |
| P14659 | Heat shock-related 70 kDa protein 2 | 691 | 1.39 |
| P20759 | Ig gamma-1 chain C region | 31 | 1.39 |
| Q07936 | Annexin A2 | 474 | 1.38 |
| P10719 | ATP synthase subunit beta_ mitochondrial | 87 | 1.38 |
| P63018 | Heat shock cognate 71 kDa protein | 852 | 1.36 |
| Q5XI73 | Rho GDP-dissociation inhibitor 1 | 327 | 1.36 |
| P21807 | Peripherin | 195 | 1.35 |
| Q68FY0 | Cytochrome b-c1 complex subunit 1_ mitochondrial | 160 | 1.35 |
| Q64122 | Myosin regulatory light polypeptide 9 | 4053 | 1.34 |
| P63102 | 14-3-3 protein zeta/delta | 142 | 1.34 |
| P68511 | 14-3-3 protein eta | 63 | 1.34 |
| O88989 | Malate dehydrogenase_ cytoplasmic | 103 | 1.34 |
| P06399 | Fibrinogen alpha chain | 112 | 1.32 |
| Q9WVH8 | Fibulin-5 | 52 | 1.32 |
| Q5RKI0 | WD repeat-containing protein 1 | 192 | 1.32 |
| Q9QXQ0 | Alpha-actinin-4 | 431 | 1.31 |
| P55063 | Heat shock 70 kDa protein 1-like | 597 | 1.31 |
| P85973 | Purine nucleoside phosphorylase | 155 | 1.31 |
| P23565 | Alpha-internexin | 69 | 1.31 |
| Q9Z1P2 | Alpha-actinin-1 | 719 | 1.30 |
| P0DMW1 | Heat shock 70 kDa protein 1B | 845 | 1.30 |
| P16409 | Myosin light chain 3 | 787 | 1.30 |
| P0DMW0 | Heat shock 70 kDa protein 1A | 849 | 1.28 |
| P02600 | Myosin light chain 1/3_ skeletal muscle isoform | 809 | 1.28 |
| P18666 | Myosin regulatory light chain 12B | 975 | 1.27 |
| P70490 | Lactadherin | 112 | 1.27 |
| P48675 | Desmin | 467 | 1.26 |
| P13832 | Myosin regulatory light chain RLC-A | 975 | 1.26 |
| P05065 | Fructose-bisphosphate aldolase A | 202 | 1.26 |
| P14668 | Annexin A5 | 145 | 1.25 |
| P31000 | Vimentin | 1733 | 1.23 |
| P06761 | Endoplasmic reticulum chaperone BiP | 248 | 1.22 |
| Q7M0E3 | Destrin | 1706 | 1.22 |
| P09495 | Tropomyosin alpha-4 chain | 50 | 1.22 |
| P01836 | Ig kappa chain C region_ A allele | 701 | 1.22 |
| P15800 | Laminin subunit beta-2 | 47 | 1.22 |
| P62963 | Profilin-1 | 3064 | 1.19 |
| P02680 | Fibrinogen gamma chain | 682 | 1.19 |
| P20761 | Ig gamma-2B chain C region | 130 | 1.19 |
| P04692 | Tropomyosin alpha-1 chain | 736 | 1.19 |
| P07943 | Aldose reductase | 254 | 1.17 |
| P58775 | Tropomyosin beta chain | 736 | 1.17 |
| Q63610 | Tropomyosin alpha-3 chain | 50 | 1.17 |
| P13437 | 3-ketoacyl-CoA thiolase_ mitochondrial | 85 | 1.16 |
| P48037 | Annexin A6 | 120 | 1.15 |
| Q08163 | Adenylyl cyclase-associated protein 1 | 165 | 1.15 |
| P24090 | Alpha-2-HS-glycoprotein | 275 | 1.15 |
| P12346 | Serotransferrin | 1002 | 1.14 |
| P20760 | Ig gamma-2A chain C region | 643 | 1.13 |
| P04797 | Glyceraldehyde-3-phosphate dehydrogenase | 1896 | 1.13 |
| Q6IE24 | Inactive ubiquitin carboxyl-terminal hydrolase 54 | 36 | 1.13 |
| P60711 | Actin_ cytoplasmic 1 | 4354 | 1.12 |
| P63259 | Actin_ cytoplasmic 2 | 4358 | 1.12 |
| P01026 | Complement C3 | 57 | 1.12 |
| P02770 | Serum albumin | 7160 | 1.09 |
| Q64119 | Myosin light polypeptide 6 | 13395 | 1.09 |
| P61983 | 14-3-3 protein gamma | 53 | 1.09 |
| P62260 | 14-3-3 protein epsilon | 148 | 1.09 |
| P14480 | Fibrinogen beta chain | 941 | 1.08 |
| P68255 | 14-3-3 protein theta | 164 | 1.08 |
| P68035 | Actin_ alpha cardiac muscle 1 | 5542 | 1.07 |
| P68136 | Actin_ alpha skeletal muscle | 5242 | 1.07 |
| P62738 | Actin_ aortic smooth muscle | 5534 | 1.07 |
| P63269 | Actin_ gamma-enteric smooth muscle | 5534 | 1.07 |
| P07632 | Superoxide dismutase [Cu-Zn] | 125 | 1.07 |
| P45592 | Cofilin-1 | 297 | 1.06 |
| P04764 | Alpha-enolase | 138 | 1.05 |
| P11884 | Aldehyde dehydrogenase_ mitochondrial | 90 | 1.05 |
| P35213 | 14-3-3 protein beta/alpha | 164 | 1.03 |
| P20059 | Hemopexin | 409 | 1.02 |
| P51886 | Lumican | 1267 | 1.00 |
| P04906 | Glutathione S-transferase P | 114 | 0.95 |
| P62982 | Ubiquitin-40S ribosomal protein S27a | 274 | 0.93 |
| Q63429 | Polyubiquitin-C | 274 | 0.92 |
| P12839 | Neurofilament medium polypeptide | 79 | 0.92 |
| Q66HD0 | Endoplasmin | 51 | 0.90 |
| P0CG51 | Polyubiquitin-B | 274 | 0.90 |
| P62986 | Ubiquitin-60S ribosomal protein L40 | 274 | 0.90 |
| P06866 | Haptoglobin | 335 | 0.88 |
| Q63598 | Plastin-3 | 90 | 0.86 |
| P11517 | Hemoglobin subunit beta-2 | 3388 | 0.86 |
| P02091 | Hemoglobin subunit beta-1 | 10419 | 0.76 |
| Q68FU3 | Electron transfer flavoprotein subunit beta | 536 | 0.68 |
| P01946 | Hemoglobin subunit alpha-1/2 | 14931 | 0.62 |
| P26772 | 10 kDa heat shock protein_ mitochondrial | 416 | SHR_P_* |
| Q5XI78 | 2-oxoglutarate dehydrogenase_ mitochondrial | 90 | SHR_P_ |
| P17764 | Acetyl-CoA acetyltransferase_ mitochondrial | 180 | SHR_P_ |
| Q5XIK1 | Actin-related protein T1 | 73 | SHR_P_ |
| P11030 | Acyl-CoA-binding protein | 271 | SHR_P_ |
| P17475 | Alpha-1-antiproteinase | 149 | SHR_P_ |
| P10759 | AMP deaminase 1 | 101 | SHR_P_ |
| Q5M9H0 | Ankyrin repeat and SAM domain-containing protein 3 | 98 | SHR_P_ |
| D3ZAF6 | ATP synthase subunit f_ mitochondrial | 55 | SHR_P_ |
| Q925T8 | BMP/retinoic acid-inducible neural-SHRPecific protein 1 | 95 | SHR_P_ |
| P0DP29 | Calmodulin-1 | 145 | SHR_P_ |
| P0DP30 | Calmodulin-2 | 145 | SHR_P_ |
| P0DP31 | Calmodulin-3 | 145 | SHR_P_ |
| Q08290 | Calponin-1 | 92 | SHR_P_ |
| P18418 | Calreticulin | 109 | SHR_P_ |
| P24268 | Cathepsin D | 117 | SHR_P_ |
| Q5U3Z0 | Cilia- and flagella-associated protein 298 | 158 | SHR_P_ |
| P11442 | Clathrin heavy chain 1 | 71 | SHR_P_ |
| P05371 | Clusterin | 93 | SHR_P_ |
| Q4KM47 | Cyclin-dependent kinase 10 | 102 | SHR_P_ |
| P14841 | Cystatin-C | 264 | SHR_P_ |
| P11240 | Cytochrome c oxidase subunit 5A_ mitochondrial | 587 | SHR_P_ |
| P10818 | Cytochrome c oxidase subunit 6A1_ mitochondrial | 631 | SHR_P_ |
| Q62871 | Cytoplasmic dynein 1 intermediate chain 2 | 109 | SHR_P_ |
| Q5BK18 | Cytosolic iron-sulfur assembly component 3 | 92 | SHR_P_ |
| Q8K5A9 | Death domain-containing membrane protein NRADD | 70 | SHR_P_ |
| P06214 | Delta-aminolevulinic acid dehydratase | 75 | SHR_P_ |
| Q62952 | Dihydropyrimidinase-related protein 3 | 103 | SHR_P_ |
| Q62967 | Diphosphomevalonate decarboxylase | 95 | SHR_P_ |
| Q66HC9 | Dynein intermediate chain 2_ axonemal | 95 | SHR_P_ |
| Q00911 | Early growth response protein 4 | 140 | SHR_P_ |
| P13803 | Electron transfer flavoprotein subunit alpha_ mitochondrial | 189 | SHR_P_ |
| P05197 | Elongation factor 2 | 108 | SHR_P_ |
| P14604 | Enoyl-CoA hydratase_ mitochondrial | 46 | SHR_P_ |
| Q5RKI1 | Eukaryotic initiation factor 4A-II | 92 | SHR_P_ |
| P50609 | Fibromodulin | 125 | SHR_P_ |
| Q9WUH4 | Four and a half LIM domains protein 1 | 413 | SHR_P_ |
| P10860 | Glutamate dehydrogenase 1_ mitochondrial | 253 | SHR_P_ |
| Q99MZ4 | Glutathione hydrolase 7 | 121 | SHR_P_ |
| Q63406 | Guanine nucleotide exchange factor DBS | 61 | SHR_P_ |
| P10824 | Guanine nucleotide-binding protein G(i) subunit alpha-1 | 104 | SHR_P_ |
| P04897 | Guanine nucleotide-binding protein G(i) subunit alpha-2 | 157 | SHR_P_ |
| P08753 | Guanine nucleotide-binding protein G(k) subunit alpha | 104 | SHR_P_ |
| P59215 | Guanine nucleotide-binding protein G(o) subunit alpha | 104 | SHR_P_ |
| P38406 | Guanine nucleotide-binding protein G(olf) subunit alpha | 104 | SHR_P_ |
| P63095 | Guanine nucleotide-binding protein G(s) subunit alpha isoforms short | 104 | SHR_P_ |
| Q63803 | Guanine nucleotide-binding protein G(s) subunit alpha isoforms XLas | 104 | SHR_P_ |
| P29348 | Guanine nucleotide-binding protein G(t) subunit alpha-3 | 104 | SHR_P_ |
| Q63210 | Guanine nucleotide-binding protein subunit alpha-12 | 101 | SHR_P_ |
| Q6Q7Y5 | Guanine nucleotide-binding protein subunit alpha-13 | 101 | SHR_P_ |
| Q9Z136 | Hamartin | 51 | SHR_P_ |
| P62959 | Histidine triad nucleotide-binding protein 1 | 223 | SHR_P_ |
| Q99MK2 | Histone acetyltransferase KAT5 | 156 | SHR_P_ |
| Q00729 | Histone H2B type 1-A | 1001 | SHR_P_ |
| Q9ESM2 | Hyaluronan and proteoglycan link protein 2 | 59 | SHR_P_ |
| P20762 | Ig gamma-2C chain C region | 127 | SHR_P_ |
| P01835 | Ig kappa chain C region_ B allele | 2149 | SHR_P_ |
| Q99NA5 | Isocitrate dehydrogenase [NAD] subunit alpha_ mitochondrial | 82 | SHR_P_ |
| Q6IFW6 | Keratin_ type I cytoskeletal 10 | 74 | SHR_P_ |
| P25030 | Keratin_ type I cytoskeletal 20 | 65 | SHR_P_ |
| P70615 | Lamin-B1 | 53 | SHR_P_ |
| Q99MZ8 | LIM and SH3 domain protein 1 | 97 | SHR_P_ |
| Q5XI07 | Lipoma-preferred partner homolog | 326 | SHR_P_ |
| A1A5P5 | LisH domain-containing protein ARMC9 | 128 | SHR_P_ |
| P04642 | L-lactate dehydrogenase A chain | 122 | SHR_P_ |
| P42123 | L-lactate dehydrogenase B chain | 214 | SHR_P_ |
| P30904 | Macrophage migration inhibitory factor | 169 | SHR_P_ |
| P02761 | Major urinary protein | 48 | SHR_P_ |
| O35763 | Moesin | 109 | SHR_P_ |
| P16884 | Neurofilament heavy polypeptide | 96 | SHR_P_ |
| P19527 | Neurofilament light polypeptide | 89 | SHR_P_ |
| O08658 | Nuclear pore complex protein Nup88 | 54 | SHR_P_ |
| Q05982 | Nucleoside diphosphate kinase A | 480 | SHR_P_ |
| P19804 | Nucleoside diphosphate kinase B | 447 | SHR_P_ |
| Q63371 | P2Y purinoceptor 6 | 95 | SHR_P_ |
| P27657 | Pancreatic triacylglycerol lipase | 58 | SHR_P_ |
| P52944 | PDZ and LIM domain protein 1 | 213 | SHR_P_ |
| Q66HS7 | PDZ and LIM domain protein 3 | 128 | SHR_P_ |
| Q62920 | PDZ and LIM domain protein 5 | 93 | SHR_P_ |
| Q9Z1Z9 | PDZ and LIM domain protein 7 | 118 | SHR_P_ |
| Q63716 | Peroxiredoxin-1 | 105 | SHR_P_ |
| Q9Z221 | Polyamine-modulated factor 1-binding protein 1 | 106 | SHR_P_ |
| D4ABH7 | Pre-miRNA 5'-monophosphate methyltransferase | 148 | SHR_P_ |
| P04785 | Protein disulfide-isomerase | 91 | SHR_P_ |
| Q9QZQ5 | Protein NOV homolog | 256 | SHR_P_ |
| P05964 | Protein S100-A6 | 1473 | SHR_P_ |
| P98106 | P-selectin | 57 | SHR_P_ |
| P49432 | Pyruvate dehydrogenase E1 component subunit beta_ mitochondrial | 53 | SHR_P_ |
| P29315 | Ribonuclease inhibitor | 58 | SHR_P_ |
| P57760 | Serine/threonine-protein kinase 16 | 110 | SHR_P_ |
| Q9QWN8 | Spectrin beta chain_ non-erythrocytic 2 | 54 | SHR_P_ |
| G3V7P1 | Syntaxin-12 | 92 | SHR_P_ |
| Q63635 | Syntaxin-6 | 91 | SHR_P_ |
| Q5XHX6 | Thioredoxin domain-containing protein 2 | 55 | SHR_P_ |
| Q6IE14 | Transmembrane protease serine 11B-like protein | 106 | SHR_P_ |
| P02767 | Transthyretin | 216 | SHR_P_ |
| P27435 | Tryptase | 591 | SHR_P_ |
| Q9EQT5 | Tubulointerstitial nephritis antigen-like | 310 | SHR_P_ |
| Q498R7 | UPF0587 protein C1orf123 homolog | 79 | SHR_P_ |
| Q6AY86 | Vacuolar protein sorting-associated protein 26A | 104 | SHR_P_ |
| O54975 | Xaa-Pro aminopeptidase 1 | 107 | SHR_P_ |
| P80299 | Bifunctional epoxide hydrolase 2 | 47 | SHR_C_* |
| Q4V8E4 | Cilia- and flagella-associated protein 36 | 83 | SHR_C_ |
| O55096 | Dipeptidyl peptidase 3 | 45 | SHR_C_ |
| Q91XQ4 | DNA-directed RNA polymerase II subunit GRINL1A | 69 | SHR_C_ |
| Q62862 | Dual specificity mitogen-activated protein kinase kinase 5 | 67 | SHR_C_ |
| Q5EZ72 | Ectonucleotide pyrophosphatase/phosphodiesterase family member 7 | 42 | SHR_C_ |
| Q66H04 | F-box only protein 43 | 68 | SHR_C_ |
| P13255 | Glycine N-methyltransferase | 44 | SHR_C_ |
| Q5XHZ0 | Heat shock protein 75 kDa_ mitochondrial | 47 | SHR_C_ |
| P06762 | Heme oxygenase 1 | 60 | SHR_C_ |
| E9PU28 | Inosine-5'-monophosphate dehydrogenase 2 | 79 | SHR_C_ |
| Q99J82 | Integrin-linked protein kinase | 75 | SHR_C_ |
| P18588 | Interferon-induced GTP-binding protein Mx1 | 61 | SHR_C_ |
| P56574 | Isocitrate dehydrogenase [NADP]_ mitochondrial | 74 | SHR_C_ |
| Q5U2U7 | mRNA cap guanine-N7 methyltransferase | 99 | SHR_C_ |
| A7E3N2 | Neutrophil cytosol factor 2 | 52 | SHR_C_ |
| O88767 | Protein/nucleic acid deglycase DJ-1 | 229 | SHR_C_ |
| Q9JK11 | Reticulon-4 | 39 | SHR_C_ |
| P42346 | Serine/threonine-protein kinase mTOR | 52 | SHR_C_ |
| D3ZVU1 | SprT-like domain-containing protein Spartan | 72 | SHR_C_ |
| P01048 | T-kininogen 1 | 55 | SHR_C_ |

^a^Identification is based on proteins ID from UniProt protein database, reviewed only (<http://www.uniprot.org/>).

^b^Proteins with expression significantly altered are organized according to the ratio.

*Indicates unique proteins in alphabetical order.
